# Glutamic Acid Transporters: Targets for Neuroprotective Therapies in Parkinson’s Disease

**DOI:** 10.3389/fnins.2021.678154

**Published:** 2021-06-16

**Authors:** Xiang Li, Wenjun Wang, Jianghong Yan, Fancai Zeng

**Affiliations:** ^1^Department of Biochemistry and Molecular Biology, School of Basic Medical Science, Southwest Medical University, Luzhou, China; ^2^Institute for Cancer Medicine and School of Basic Medical Sciences, Southwest Medical University, Luzhou, China

**Keywords:** Parkinson’s disease, excitatory amino acid transporters, vesicular glutamate transporters, glutamate, glutamic acid transporters

## Abstract

Parkinson’s disease (PD) is a common neurodegenerative disease in middle-aged and elderly individuals. At present, no effective drug has been developed to treat PD. Although a variety of drugs exist for the symptomatic treatment of PD, they all have strong side effects. Most studies on PD mainly focus on dopaminergic neurons. This review highlights the function of glutamic acid transporters (GLTs), including excitatory amino acid transporters (EAATs) and vesicular glutamate transporters (VGLUTs), during the development of PD. In addition, using bioinformatics, we compared the expression of different types of glutamate transporter genes in the cingulate gyrus of PD patients and healthy controls. More importantly, we suggest that the functional roles of glutamate transporters may prove beneficial in the treatment of PD. In summary, VGLUTs and EAATs may be potential targets in the treatment of PD. VGLUTs and EAATs can be used as clinical drug targets to achieve better efficacy. Through this review article, we hope to enable future researchers to improve the condition of PD patients.

## Introduction

Parkinson’s disease (PD) is a common neurodegenerative disease characterized by resting tremor, bradykinesia, rigidity, and postural instability. Freezing of gait is a refractory symptom of PD, which is manifested by the short and sudden stop of the patient’s step when he or she tries to walk or move forward. Freezing of gait, which is common in patients in the middle and late stages of PD, seriously affects the quality of life and prognosis of these patients. In addition, PD patients can also experience depression, olfactory dysfunction, sleep disorders, cognitive impairment, fatigue, and other non-motor symptoms (Kalia and Lang, 2015), which are common in early PD. The progression of PD is characterized by deterioration of motor function, which can initially be controlled by symptomatic treatment. However, as drug therapy continues, complications, such as dyskinesia and psychosis, may arise.

Parkinson’s disease is the second most important age-related neurodegenerative disorder in developed societies, after Alzheimer’s disease (AD). It is predicted that by 2030, approximately 3% of the global population over the age of 65 may be affected (Palakurthi and Burugupally, 2019). The main pathological feature of PD is the selective loss of dopaminergic neurons in the substantia nigra (SN) and the presence of Lewy bodies in the midbrain (Blauwendraat et al., 2020). The selective vulnerability of neurons in the SN is caused by several putative mechanisms, such as dopamine metabolism (da Silva et al., 2018), increased oxidative stress (Li et al., 2020b), iron accumulation (Xu et al., 2019; Bi et al., 2020; Lee et al., 2020), mitochondrial dysfunction (Park et al., 2020; Rani and Mondal, 2020), and dysregulation of the ubiquitin–proteasome system (Zheng et al., 2016). Another pathophysiological feature of PD is the loss of glutamatergic synapses and the increase in glutamatergic nerve transmission to the striatum (Raju et al., 2008). However, the pathogenesis of PD is not fully understood. The abnormal aggregation of α-synuclein (α-SYN), neuroinflammation, autophagy, tau hyperphosphorylation, and mitochondrial dysfunction have been reported to cause PD. The accumulation of α-SYN to form Lewy bodies is considered to be the main pathogenic mechanism of PD. Studies have shown that the pathogenesis of PD is also related to glutamate (Glu) excitotoxicity (Chassain et al., 2010; Gibson et al., 2018). The excessive synthesis or release of Glu and the reduction in Glu reuptake lead to a high concentration of Glu in the synaptic space resulting in toxicity and neuronal death.

Glu is the major excitatory neurotransmitter in the mammalian central nervous system (CNS), where it participates in the physiological regulation of different processes. It has been demonstrated that excessive endogenous Glu is related to many chronic and acute neurodegenerative disorders such as epilepsy (Levite et al., 2020), amyotrophic lateral sclerosis (Mishra et al., 2016), cerebral ischemia (Wang et al., 2013), AD (Park et al., 2019), and PD (Tamano et al., 2019). Therefore, Glu excitotoxicity plays an important role in the development of PD. The subthalamic nucleus (STN) projects to the SN pars compacta (SNpc) and SN pars reticulata (SNpr). The major cause of PD is the loss of dopaminergic neurons in the SNpc. Once these dopaminergic neurons are lost, calcium influx is induced by Glu release, which results in a cascade of reactions triggered by Ca^2+^ overload. Eventually, this process leads to degeneration and necrosis of dopaminergic neurons (Chu et al., 2017). At the same time, the reduced production of dopamine weakens the inhibition of STN and enhances the discharge of excitatory efferent neurons, which further aggravates Glu excitotoxicity (Blandini et al., 2000). Therefore, Glu neurotoxicity plays an important role in PD. To date, there are two main directions regarding the role of Glu in PD—Glu receptors and glutamic acid transporters (GLTs). Glu receptors, which are primarily located on postsynaptic and presynaptic neurons in almost all areas of the CNS, play a critical role in brain function. Pharmacologically, Glu receptors were originally classified into two major types—ionotropic Glu receptors (iGluRs) and metabotropic Glu receptors (mGluRs). iGluRs are multimeric ion channels that are responsible for fast excitatory transmission in the CNS. iGluRs transmit excitatory signals to postsynaptic neurons by binding to Glu released from presynaptic neurons. Glu can activate three types of iGluRs, namely, α-amino-3-hydroxy-5-methyl-4-isoxazolepropionic acid (AMPA) receptors, *N*-methyl-D-aspartate (NMDA) receptors, and kainate receptors. mGluRs are members of the G-protein receptor superfamily; hence, they mediate the slow Glu responses, which contribute to long-lasting changes in synaptic activity (Schapira, 2010). According to their signal transduction mechanisms, pharmacology, and sequence homology, eight different types of mGluRs are classified into three groups (Group I: mGluR1 and mGluR5, Group II: mGluR2 and mGluR3, Group III: mGluR4, mGluR6, mGluR7, and mGluR8) (Menard and Quirion, 2012). In the pathophysiology of PD, Glu receptors can undergo changes during the disease process, such as increased binding of Glu to NMDA receptors and decreased mGlu2/3 receptor expression, in monkeys (Jourdain et al., 2015). Many studies have shown that mGluRs and iGluRs play a critical role in MPTP-induced PD (Betarbet et al., 2000; Kintz et al., 2013; Al-Sweidi et al., 2016; Nuzzo et al., 2019; Mann et al., 2020). Therefore, some Glu receptor antagonists, such as amantadine (Hubsher et al., 2012) and memantine (Olivares et al., 2012), have been used as therapeutic drugs for PD. GLTs have been confirmed to have two types of transporters: excitatory amino acid transporters (EAATs) and vesicular Glu transporters (VGLUTs) (Wang et al., 2019). The level of extracellular Glu is regulated by high-affinity EAATs (Hayashi, 2018). EAATs maintain the dynamic equilibrium of the extracellular Glu concentration by protecting neurons from detrimental overstimulation of Glu receptors (Zerangue and Kavanaugh, 1996). EAATs can be divided into five subtypes: EAAT1, EAAT2, EAAT3, EAAT4, and EAAT5. In addition to the above mechanisms, VGLUT specifically transfers Glu into the synaptic vesicle, which determines the amount of Glu released into the synaptic cleft (Schenck et al., 2009). The VGLUT family presents distinct expression patterns. VGLUT1 and VGLUT2 are specifically expressed on glutamatergic neurons (El Mestikawy et al., 2011; Zhang et al., 2018a), while VGLUT3 usually acts as a cotransporter of Glu or other neurotransmitters, including gamma-aminobutyric acid (GABA), acetylcholine, and serotonin (El Mestikawy et al., 2011). Therefore, a comprehensive understanding of the role of Glu transporters in the pathophysiology of PD and as therapeutic targets will help in the development of new methods for the treatment of PD. In this review, we highlight the function of these different subtypes of Glu transporters and their potential neuroprotective effects, as well as evidence of the pharmacological manipulation of these transporters in PD.

## Molecular Mechanism of Glu Transporters in PD

Most studies have focused on the distribution, structure, and complex subunits of Glu receptors, as well as the functions of these receptors in PD. However, few studies have reported the role of EAATs and VGLUTs in regulating Glu concentration in PD.

### EAAT1

**FIGURE 1:**
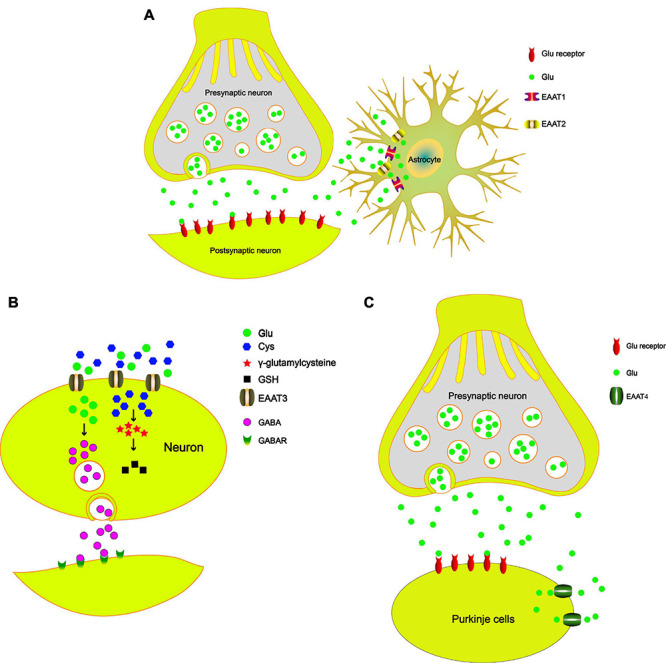
The role of EAATs in the PD model. **(A)** The tripartite synapse is composed of the presynaptic neuron, postsynaptic neuron, and astrocytes. EAAT1 and EAAT2 uptake Glu from the synaptic cleft to maintain Glu homeostasis and prevent the excitotoxicity of neurons. **(B)** EAAT3 uptakes Cys and then synthesizes GSH, which reduces oxidative damage. EAAT3 modulates GABA synthesis and maintains GABA homeostasis. **(C)** EAAT4 plays a key role in the synaptic activity of cerebellar Purkinje cells by regulating extracellular Glu concentrations.

High levels of extracellular Glu are associated with excitatory neuronal cell death. The best way to maintain the Glu concentration is to remove Glu from synapses by GLTs in astrocytes after pulsed transmission (Jia et al., 2015; Karki et al., 2015b). Upregulation of EAAT1 is related to improvements in neurological function after middle cerebral artery occlusion in rats (Matsuura et al., 2002; Mori et al., 2004). Some evidence indicates that EAAT1 functional upregulation can reduce neurotoxicity in related brain regions. Li et al. (2020a) showed that upregulation of EAAT1 may be a potential mechanism by which dopaminergic neurons are protected *via* reducing the toxicity of excitatory amino acids in a rodent model of PD. In addition, Chung et al. (2008) reported that reductions in EAAT1 can disrupt Glu homeostasis around glutamatergic synapses in the striatum and result in overspills of Glu in the CNS. This will eventually lead to damage of dopaminergic neurons. Astrocytes help to protect neurons through a variety of mechanisms, one of which is the uptake of Glu to prevent excitotoxicity (Sattler and Rothstein, 2006). Johnson et al. (2018) reported that regulation of EAAT1 protein and mRNA levels can result in resistance to manganese-induced neurotoxicity in mice. In other words, EAAT1 regulates the levels of Glu to resist neurotoxicity, including that which occurs in PD. Another study also showed that upregulating gene and protein expression levels of EAAT1 in cultured astrocytes could offer neuroprotection from excitotoxicity (Neal et al., 2018). Wu et al. (2019) found that upregulation EAAT1 triggers the Wnt signaling pathway and promotes dopaminergic neuron proliferation, thereby suppressing apoptosis in PD mice. In the acute MPTP (1-methyl-4-phenyl-1,2,3,6-tetrahydropyridine) mouse model and 6-hydroxydopamine (6-OHDA)-lesioned rat model of PD, decreased immunoreactivity and gene expression levels of EAAT1 are observed in the striatum (Holmer et al., 2005; El Arfani et al., 2015). Taken together, these data indicate that PD can lead to functional changes in astrocytes, which in turn lead to increased extracellular Glu through downregulation of the Glu transporter EAAT1, thereby damaging dopaminergic neurons and causing neurotoxicity. EAAT1 transports Glu from the synaptic cleft to maintain Glu homeostasis and prevent neuronal excitotoxicity (Figure 1A). Therefore, regulation of EAAT1 expression may be a potential therapeutic target in PD.

### EAAT2

A series of studies has reported that EAAT2 is responsible for approximately 80% of total extracellular Glu uptake activities (Danbolt et al., 1992; Haugeto et al., 1996; Tanaka et al., 1997; Holmseth et al., 2012). Similar to EAAT1, EAAT2 is also primarily expressed in astrocytes. Reduced expression and function of EAAT2 have been reported in various neurodegenerative disorders, including AD and PD (Lin et al., 2012; Karki et al., 2015a). Many studies have reported that the Wnt signaling pathway can induce the EAAT2 expression (Palos et al., 1999; Parkin et al., 2018). A previous study showed that nuclear factor-κB (NF-κB) contributes to the neuron-dependent induction of EAAT2 expression (Ghosh et al., 2011). Many studies have shown that NF-κB is involved in the protective effects of the Wnt signaling pathway (Bournat et al., 2000; Shang et al., 2010). Moreover, Wei et al. (2019) demonstrated that Wnt regulates the EAAT2 level in astrocytes and protects dopaminergic cells against 6-OHDA toxicity. Zhang et al. (2018b) showed that ginsenoside could increase EAAT2 expression *via* NF-κB. Upregulation of EAAT2 can terminate Glu excitotoxicity and improve PD. These studies indicate that cross talk between the Wnt signaling pathway and the NF-κB pathway participates in the regulation of EAAT2 expression. Recently, many studies reported that the protein levels of EAAT2 in the striatum are decreased in animal models of PD according to immunofluorescence and western blot analyses, including in the 6-OHDA-lesioned PD model (Chung et al., 2008) and the MPTP-treated mouse model (Holmer et al., 2005). Chotibut et al. (2017) demonstrated that l-3,4-dihydroxyphenylalanine (L-dopa)-induced dyskinesia severity is reduced when the expression of EAAT2 is increased in a rat 6-OHDA model of PD. Zhang et al. (2020) found that EAAT2 deficiency in the SN induces a PD phenotype according to progressive motor deficits and the death of nigral dopaminergic neurons in mice. Singh et al. (2018) found that acetyl-L-carnitine could reduce the accumulation of ROS and promote the expression of EAAT2 to protect neurons. In addition, acetyl-L-carnitine treatment protects DA neurons, thus preserving the integrity of the nigrostriatal pathway, which may result in improvement in PD. Ceftriaxone can increase EAAT2 expression and decrease Glu content to protect neurons. In addition, Leung et al. (2012) demonstrated that the side chain of ceftriaxone, D-α-aminoadipic acid, is readily carbonylated upon oxidative damage and prevents the carbonylation of endogenous target, thereby protecting neurons (Tikka et al., 2001). All these studies suggest that EAAT2 is an important target for the treatment of PD. EAAT2 transports Glu from the synaptic cleft to maintain Glu homeostasis and prevent neuronal excitotoxicity (Figure 1A). Therefore, some well-known drugs protect dopaminergic neurons by upregulating the expression of EAAT2, which is an important mechanism for the treatment and prevention of PD.

### EAAT3

EAAT3 is primarily expressed in neurons, especially at postsynaptic terminals (He and Casaccia-Bonnefil, 2008). Even though EAAT3 is expressed in all CNS neurons (Rothstein et al., 1994; Shashidharan et al., 1997), growing evidence indicates that EAAT3 expression is particularly dense on SN dopaminergic neurons (Nafia et al., 2008; Berman et al., 2011). Aoyama found that EAAT3 is involved in MPTP-induced glutathione depletion and motor dysfunction (Aoyama et al., 2008). In addition to Glu, EAAT3 also mediates the transport of cysteine (Cys) in neurons (Hayes et al., 2005), where it provides a Cys substrate for the synthesis of glutathione (GSH). Consistent with this view, EAAT3 inhibition leads to GSH loss and subsequent cell death of dopaminergic neurons in mice and rats (Nafia et al., 2008). Therefore, deletion of EAAT3 in mice impairs neuronal Cys uptake, which results in chronic neuronal oxidative stress. Berman et al. (2011) suggested that oral N-acetylcysteine could restore reactive thiol content in EAAT3 knockout dopaminergic neurons. In addition to transporting Glu and Cys, EAAT3 also plays an important role in regulating GABA (γ-aminobutyric acid) synthesis (Sepkuty et al., 2002), reducing oxidative stress in neurons (Yang et al., 2018), and supporting neuron viability (Duerson et al., 2009). Downregulation of EAAT3 expression through antisense RNA approaches increases the sensitivity of neurons to excitotoxic injury (Brustovetsky et al., 2004) and causes dendritic swelling in the hippocampus (Rothstein et al., 1996). More importantly, recent studies have indicated that EAAT3 deficiency leads to neuronal loss (Aoyama et al., 2006), which shows that EAAT3 is essential to the viability of supporting neurons. Increasing evidence suggests that EAAT3 can modulate GABA synthesis (Mathews and Diamond, 2003) and that the levels of GABA are crucial to PD development (Blaszczyk, 2016; Firbank et al., 2018; Liu et al., 2019). These results support the view that EAAT3 dysfunction can lead to a range of damaging neuropathological consequences, including decreased GABA synthesis, increased susceptibility to oxidative neuronal injury, decreased Glu clearance, and ultimately neuronal loss. Exercise can alleviate locomotor impairment in PD. Arnold and Salvatore (2016) demonstrated that the expression of EAAT3 and tyrosine hydroxylase (TH) is increased after short-term exercise. Together, these findings suggest an important role for EAAT3 in dopaminergic neuronal GSH metabolism and a contributory role for GSH depletion in dopaminergic neuronal death. In addition, EAAT3 also influences the levels of GABA, which affects the development of parkinsonian symptoms. In summary, EAAT3 can transport Cys and synthesize GSH, which reduces oxidative damage. EAAT3 can modulate GABA synthesis and maintain GABA homeostasis, which has an important role in the development of PD (Figure 1B).

### EAAT4

EAAT4 is a member of the high-affinity Na^+^/K^+^-dependent Glu transporter family and is mainly expressed in Purkinje cells of the cerebellum (Massie et al., 2008). EAAT4 plays a key role in the synaptic activity of cerebellar Purkinje cells by regulating extracellular Glu concentrations (Jiang et al., 2016). Takada et al. (1993) reported that neuronal degeneration induced by MPTP occurs in the cerebellum and is characterized by the loss of Nissl-stained Purkinje cells. The STN projects to the cerebellum likely by way of the pontine nuclei (PN) (Bostan and Strick, 2018), and the projection from the PN to the cerebellum consists of glutamatergic neurons (Beitz et al., 1986). A previous study suggested that midbrain dopaminergic neurons affect glutamatergic transmission in the cerebellum, which may be important for the generation of the tremors (Golembiowska et al., 2013). In a rat model of PD induced by the intrastriatal injection of rotenone, the concentration of Glu in the cerebellum is significantly increased (Khadrawy et al., 2017). In addition, Zhou et al. (2018) reported that miR-128 overexpression contributes to EAAT4 upregulation and protects dopaminergic neurons from apoptosis, thereby alleviating PD in mice. Therefore, according to current research, Glu neurotransmitters should be considered during the treatment of PD. To our knowledge, the roles of the cerebellum in PD remain limited, and further focus on the cerebellum should be centered on Glu transporters, especially EAAT4. In summary, we propose that in Purkinje cells of the cerebellum, EAAT4 influences the development of parkinsonian symptoms (such as bradykinesia, rigidity, and tremor) by regulating the Glu concentration (Figure 1C).

### EAAT5

EAAT5 is primarily expressed in bipolar cells, photoreceptors, and amacrine cells. EAAT5 is a well-known chloride channel activated by Glu that controls the excitability of retinal neurons (Picaud et al., 1995; Arriza et al., 1997; Eliasof et al., 1998; Pow and Barnett, 2000; Wersinger et al., 2006). EAAT5 has the largest chloride conductance than the other types of EAATs, and its function is more as an inhibitory Glu receptor than as a transporter (Zhou and Danbolt, 2013). The physiological role of EAAT5 is not fully understood, but its low Glu transport rate and Glu affinity argue against a major role in Glu reuptake, and EAAT5 is always considered a Glu-gated anion channel. To date, no animal models deficient in EAAT5 have been reported, and no disease has been linked to gene mutations in EAAT5 in humans. Therefore, the development of these models may enable us to clearly identify the cellular function of EAAT5. The particularity of EAAT5 may be one reason for the lack of reports that describe a relationship between EAAT5 and PD.

### VGLUT1

**FIGURE 2:**
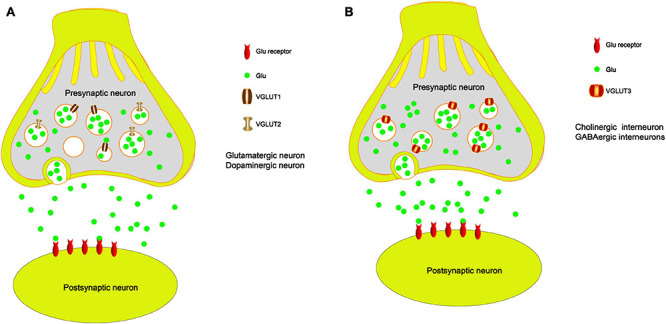
The role of VGLUTs in the PD model. **(A)** VGLUT1 and VGLUT2 load Glu into vesicles, and they fuse with the presynaptic membrane and release Glu into the synaptic cleft, especially in the glutamatergic neurons and dopaminergic neurons. **(B)** VGLUT3 loads Glu into vesicles, and it fuses with the presynaptic membrane and releases Glu into the synaptic cleft, especially in the cholinergic neurons and GABAergic neurons.

Vesicular glutamate transporters are located in the membrane of presynaptic glutamatergic synaptic vesicles and are responsible for loading Glu into vesicles (Figure 2A). Then, the vesicles fuse with the presynaptic membrane and releases Glu into the synaptic cleft (Massie et al., 2010). Since the number of VGLUT molecules in each synaptic vesicle plays an important role in the quantal size of glutamatergic neurons, changes in VGLUT expression may have a significant impact on synaptic transmission (Wojcik et al., 2004). The expression of VGLUTs is an indicator of the relative synaptic strength of presynaptic glutamatergic innervation in specific brain regions (Massie et al., 2010). Therefore, VGLUTs play an important role in neurodegenerative diseases (Daniels et al., 2011; Steinkellner et al., 2018; Ibrahim et al., 2020). VGLUT1 is one of the specific markers of glutamatergic neurons. A series of studies has shown that VGLUT1 is expressed in the hippocampus, thalamus, cerebral, cerebellar cortices, amygdala, and cerebellum (Ni et al., 1995; Bellocchio et al., 1998; Herzog et al., 2001; Lee et al., 2015; Fattorini et al., 2017; Fang et al., 2018). Many studies have shown that the protein expression of VGLUT1 is reduced in the prefrontal cortex (PFC) of PD patients compared with control subjects (Kashani et al., 2007; Granseth et al., 2015). However, Raju et al. (2008) found that the total density of VGLUT1 is significantly increased in the striatum of MPTP-treated parkinsonian monkeys. In addition, postmortem human data also support the view that VGLUT1 expression is increased in the striatum of PD patients (Kashani et al., 2007). A previous study found that complete knockout of VGLUT1 leads to postnatal lethality in C57BL/6 mice and that heterozygous mice show impaired hippocampal long-term potentiation (LTP) accompanied by a deficit in spatial reversal learning (Balschun et al., 2010). Fremeau et al. (2004) showed that synaptic vesicle-enriched membrane fractions from VGLUT1–/– mice also exhibit a reduction in Glu. Deletion of VGLUT most likely alters the probability of Glu release from nerve endings. Clinically, the STN is an important target of deep brain electrical stimulation in the treatment of PD. Wang showed that electroacupuncture treatment restores the downregulation of VGLUT1 in the STN in a model of PD induced by 6-OHDA (Wang et al., 2018). Electroacupuncture can reduce the motor symptoms of PD by upregulating VGLUT1 expression in the STN, which suggests that upregulation of VGLUT1 in the STN improves dyskinesia in PD through the cortico-STN pathway (Massie et al., 2010). In summary, the abnormal transmission of Glu in PD can result in disease-related motor and cognitive disorders. Increasing evidence suggests that changes in VGLUT1 protein expression are involved in the progression of PD.

### VGLUT2

Damage to DA neurons is the major cause of PD. TH is the rate-limiting enzyme in dopamine synthesis and is a marker of dopamine. Recent studies have shown that VGLUT2 and TH are coexpressed in the striatum of rats and mice (Berube-Carriere et al., 2009; Chuhma et al., 2009; Trudeau et al., 2014; Morales and Margolis, 2017). VGLUT2 is a major subtype of VGLUTs expressed in midbrain dopaminergic neurons (Morales and Root, 2014) and is responsible for loading Glu into vesicles (Figure 2A). A previous study showed that selective knockout of VGLUT2 can significantly reduce the expression of the brain-derived neurotrophic factor (BDNF) and its receptor tyrosine receptor kinase B (TrkB), which results in significant DA neuron death induced by MPTP. Rescuing the expression of VGLUT2 in DA neurons reverses these alterations (Shen et al., 2018). Steinkellner also demonstrated that overexpression of VGLUT2 is sensitive to DA neurons and that endogenous VGLUT2 deletion increases the susceptibility of DA neurons to parkinsonian neurotoxins in mice (Steinkellner et al., 2018). DA neuron loss in the SN in PD leads to overactivation of the STN. Moreover, VGLUT2 is localized in STN terminals synapsing within the SN (Moore et al., 2020). Several studies have demonstrated that VGLUT2 expression in DA neurons is necessary for normal emotional responses and psychostimulant-mediated behavioral activation (Birgner et al., 2010). In addition, a series of studies has suggested that the reduced expression levels of VGLUT2 in the STN of mice cause degeneration of midbrain DA neurons (Schweizer et al., 2014, 2016; Moore et al., 2020). Another study found that MPTP injected into mice induces a decrease in VGLUT2 expression in the striatum compared with the control group (Pflibsen et al., 2015). In other words, we suggest that VGLUT2 trafficking to sites of somatodendritic DA release may cause the activation of presynaptic Glu receptors on DA neurons, which results in excitotoxicity. These results indicated that enhanced Glu release induced by the reduced expression of VGLUT2 results in further DA neuron death. Therefore, regulating the expression of VGLUT2 in DA neurons may be an important factor in the development of PD and suggests a potential therapeutic strategy by activating DA neurons.

### VGLUT3

VGLUT3 is expressed in non-glutamatergic neurons, including cholinergic striatal interneurons, and GABAergic interneurons, which are responsible for loading Glu into vesicles (Figure 2B). VGLUT3 can also regulate DA signaling and movement activity. Therefore, VGLUT3 affects the activity of DA neurons by regulating the concentration of Glu neurotransmitters. It has been reported that VGLUT3 knockout mice exhibit hyperlocomotion (Gras et al., 2008), which is related to increased midbrain DA signaling. Divito found that deletion of VGLUT3 results in upregulation of the midbrain dopamine system in mice. The KO mice show less abnormal motor behavior after 6-OHDA administration; however, the mice present fewer abnormal dyskinesias in response to L-dopa-induced dyskinesia (Divito et al., 2015). Another study also showed that DA depletion in the striatum increases the expression levels of VGLUT3. This is mainly because the inhibitory effect of Glu on DA release is weakened. The authors also suggest that VGLUT3 plays a key role in the development of L-dopa-induced dyskinesia (Gangarossa et al., 2016). At present, few studies have demonstrated the critical role of VGLUT3 in PD. Most studies have found that VGLUT3 plays a crucial role in levodopa-induced dyskinesia, which occurs in patients with PD who have been treated with levodopa over a long period of time.

## Discussion

Parkinson’s disease is a typical neurodegenerative disease, the pathogenesis of which is complex. Previous studies have shown that Glu excitotoxicity may result in the loss of SN dopaminergic neurons (Wang et al., 2020). In PD patients, Glu excitotoxicity decreases dopaminergic neuronal transmission, while glutamatergic neurons in the basal ganglia can stimulate dopaminergic neurons in the SN to release DA as a compensatory mechanism. However, excessive activation may be lethal to dopaminergic neurons and even cause the death of these neurons (Blandini et al., 2000). No effective drug can prevent or treat PD. Judging from the current study, most drug targets mainly focus on the dopaminergic neuron system and are either dopamine replacements or dopamine receptor stimulants. Common drugs used to treat PD are classified according to their different targets, as shown in Table 1. Although various types of drugs help patients effectively alleviate Parkinson’s-like symptoms, these drugs are associated with many side effects (for example, alimentary symptoms, postural hypotension, insomnia, and dyskinesia). Currently, no drugs target EAATs or VGLUTs in the treatment of PD. Therefore, the development of Glu transporters as novel drug targets for the treatment of PD is very promising.

**TABLE 1:** Classification of common drugs for the treatment of PD.

**Type**	**Target**	**Drug**	**References**
Dopamine replacers	Dopamine neurons	L-DOPA	Armstrong and Okun, 2020
Antagonists	*N*-methyl-D-aspartic acid	Amantadine	Hubsher et al., 2012
	receptor (NMDAR)	Menantine	Olivares et al., 2012
		Riluzole	Liu and Wang, 2018
	Adenosine A2A Receptor	Istradefylline	Muller, 2015
		Tozadenant	Oertel and Schulz, 2016
Agonists	Dopamine receptors	Apomorphine	Boyle and Ondo, 2015
		Pramipexole	Frampton, 2014
		Ropinirole	Matheson and Spencer, 2000
		Piribedil	Perez-Lloret and Rascol, 2016
	Serotonin receptors	F15599	Iderberg et al., 2015
		Pimavanserin	Sahli and Tarazi, 2018
Inhibitor	Monoamine oxidase-B	Selegiline	Szoko et al., 2018
		Rasagiline	(Szoko et al., 2018
	Catechol-*O*-methyltransferase	Tolcapone	Keating and Lyseng-Williamson, 2005
		Entacapone	Schrag, 2005)
	Cholinergic neurons	Trihexyphenidyl	Takahashi et al., 1999
		Benzatropine	Agundez et al., 2013

Other neurological disorders, such as ataxia, may be relevant to the pathology of PD. An increasing body of evidence suggests that mutation in EAAT1 results in decreased uptake of Glu, which can cause hemiplegia, migraine, ataxia, and epilepsy (Jen et al., 2005; Stacey et al., 2010; Parinejad et al., 2016). In addition, in ataxia, inactivation of EAAT4 impairs spontaneous activity of Purkinje cells, which causes neuronal death (Serra et al., 2004; Perkins et al., 2010). One study showed that upregulation of EAAT4 can protect dopaminergic neurons from apoptosis, thereby alleviating PD in mice (Zhou et al., 2018). Sanjay et al. suggested that regional reductions in EAAT2-4 expression could induce increased Glu levels, which may be associated with human epilepsy (Rakhade and Loeb, 2008). Therefore, EAAT-associated anion channels may play an important role in many neurodegenerative diseases such as PD, ataxia, and epilepsy. However, the specific mechanisms remained unclear. Dopaminergic neuron death induced by oxidative stress played an important factor in PD. In addition, some EAATs act as chloride channels or mediate the uptake of Cys, which can produce the ROS scavenger GSH (Lim et al., 2005; Aoyama and Nakaki, 2013; Robert et al., 2014). EAAT also functions as an anion channel, which plays an important role in the regulation of glial cells in the CNS (Untiet et al., 2017), especially in the apoptosis of glial cells induced by ataxia (Kovermann et al., 2020). Therefore, we suggest that EAAT may have the same effect in PD. The expression levels of VGLUTs, with the exception of EAATs, determine the amount of Glu loaded into vesicles and released, thus regulating the effect of neurotransmission (Wojcik et al., 2004). Therefore, changes in the expression of VGLUTs may have a significant impact on synaptic transmission and may ultimately affect neurons. Kashani et al. (2007) suggested that the downregulation of VGLUT1 in the PFC is associated with cognitive dysfunction in PD. The mechanism may be that the level of VGLUT1 can directly affect presynaptic quantal size, which supports the possibility that increased expression of VGLUT1 can enhance corticostriatal neuron signaling (Wojcik et al., 2004; Wilson et al., 2005); this may serve as an adaptive mechanism to compensate for the loss of dopaminergic neurons. In addition, upregulation of VGLUT2 dopaminergic neurons can promote Glu release, which drives axonal outgrowth and results in dopaminergic neuron axonal plasticity in the postlesional brain (Kouwenhoven et al., 2020). We suggest that this increase promotes the survival of dopaminergic neurons. A previous study showed that deletion of VGLUT3 not only abolishes the Glu signaling (Higley et al., 2011) but also significantly reduces cholinergic transmission (Nelson et al., 2014). Therefore, these results showed that VGLUTs may play an important role in the treatment of PD by regulating the levels of acetylcholine and Glu. In addition, Daniels et al. (2006) found only one VGLUT per synaptic vesicle. In other words, upregulation of VGLUT can result in the addition of another transporter and an increase in quantal size, while downregulation of VGLUT leads to a mix of vesicles or no transporter and empty vesicles. In this way, VGLUT may be important for vesicle recycling and the rapid regulation of Glu concentration.

**FIGURE 3:**
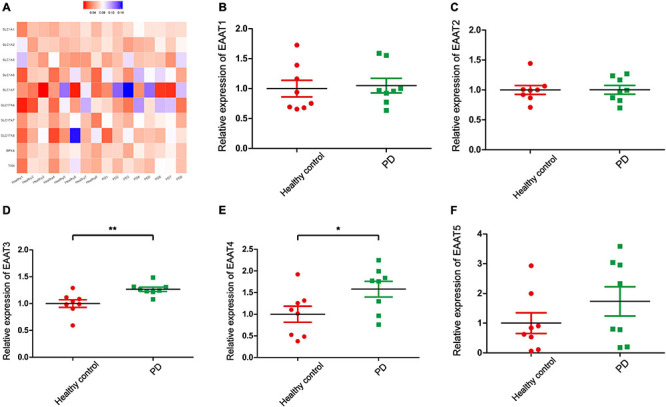
Bioinformatics analysis for gene transcriptional expression and the relative expression of EAATs. **(A)** Heatmap showed the expression of EAATs and VGLUTs in 16 samples. **(B)** The relative expression of EAAT1 in PD patients and healthy controls. **(C)** The relative expression of EAAT2 in PD patients and healthy controls. **(D)** The relative expression of EAAT3 in PD patients and healthy controls. **(E)** The relative expression of EAAT4 in PD patients and healthy controls. **(F)** The relative expression of EAAT5 in PD patients and healthy controls. Each bar represents the mean ± SE (*n* = 8). *P < 0.05, **P < 0.01, statistically significant.

**FIGURE 4:**
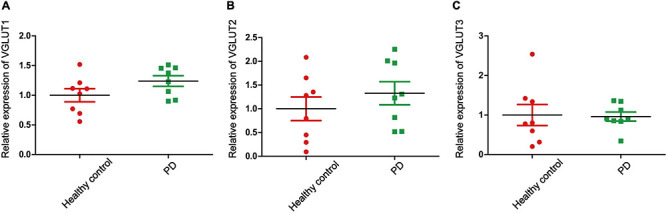
The relative expression of VGLUTs. **(A)** The relative expression of VGLUT1 in PD patients and healthy controls. **(B)** The relative expression of VGLUT2 in PD patients and healthy controls. **(C)** The relative expression of VGLUT3 in PD patients and healthy controls. Each bar represents the mean ± SE (*n* = 8).

Glu is also the major excitatory neurotransmitter in the basal ganglia, which is the site of dyskinesia in PD (Greenamyre et al., 1994). Some studies have reported that high levels of Glu in the brain lead to increased accumulation of reactive oxygen species (ROS), which leads to an imbalance in intracellular redox. In other words, ROS induced by Glu excitotoxicity play an important role in the development of PD (Son et al., 2013; Buchanan et al., 2015). Therefore, regulating the concentration of Glu in the brain may be a potential treatment for PD and recent studies have focused on the role of Glu receptors in PD. However, few studies on the role of EAATs and VGLUTs in PD have been published. In this review, we report that different types of EAATs and VGLUTs are located in different types of neurons, including dopaminergic neurons, glutamatergic neurons, cholinergic neurons, and GABAergic neurons. With the exception of EAAT5, many studies have reported that Glu transporters play an important role in the course of PD development. Increasing evidence suggests that dysregulation of the glutamatergic nervous system is one of the major reasons for disease and that these disorders accelerate disease progression. Studies on the mechanism of Glu transporters in the pathological changes of neuronal synapses, synaptic plasticity, filling size, and transport velocity of synaptic vesicles are inadequate. Regulating the function of EAATs and VGLUTs to promote neuroprotection may be an effective intervention and treatment strategy for the treatment of PD. In addition, we examined the expression of Glu transporters in the cingulate gyrus of eight patients with PD and eight control subjects. The cingulate gyrus, a region in which α-SYN accumulates, is a key node of the neural network, which includes the amygdala, the ventral striatum, and the SN, and is related to cognitive impairment (Bressler and Menon, 2010). In addition, L-6-18F-fluoro-3,4-dihydroxyphenylalanine (FDOPA) PET has been widely used to quantify dopamine metabolism in the clinic. This method is used to evaluate the ability of neurons in the SN to synthesize dopamine. The cingulate gyrus, which receives dopaminergic projections from the ventral tegmental area through the mesolimbic pathway, is the region of highest FDOPA uptake among frontal cortical regions (Kumakura et al., 2010; Blanchet and Brefel-Courbon, 2018). Therefore, exploring Glu transporters in the cingulate gyrus may be important in the early clinical diagnosis and treatment of PD. We used transcriptome datasets downloaded from NCBI (GSE110716). Heatmap showed the expression of EAATs and VGLUTs (Figure 3A). We found that the expression levels of EAAT3 and EAAT4 are upregulated in patients compared with control subjects in Figures 3D,E. In contrast, the other types of Glu transporters are not altered (Figures 3B,C,F, 4). In patients with PD, the increased levels of EAAT3 and EAAT4 may be a protective mechanism that alleviates PD symptoms. The other types of Glu transporters exhibit no significant changes, which may be due to the following reasons: 1. The number of human samples is too small. 2. The expression of Glu transporters may vary according to brain region. Importantly, we found that in human patients with PD, Parkinson’s symptoms may be alleviated by increasing the expression of Glu transporters.

Currently, an increase in the understanding of the function of Glu receptors in the nervous system is highly warranted. Future studies should include the following areas of research: upregulating or interfering with the expression of Glu transporters in different brain regions in a PD model, detecting the role of Glu transporter agonists or inhibitors in a PD model, and assessing the combined effects of different types of Glu transporters in a PD model and the combined effects of Glu transporters and other PD targets in a PD model. Although many studies have demonstrated that abnormal levels of Glu transporters may be one of the causes of various mental disorders, the origins of these complex disorders are not yet fully understood. Further understanding of the function of EAATs and VGLUTs may also prove highly beneficial to elucidating the mechanisms of PD and other nervous system diseases. In the future, we hope to improve the condition of PD patients.

## Conclusion

In this review, we highlighted the potential roles of EAATs and VGLUTs in the treatment of PD. With the exception of EAAT5, Glu transporters have significant functions in regulating the levels of Glu, DA, and GABA. Many studies have shown that Glu transporters can improve excitatory toxicity in neurons by regulating the activities of dopaminergic neurons, glutamatergic neurons, and GABAergic neurons in the development of PD. In addition, by regulating the expression of Glu transporters, the levels of ROS in neurons can be affected. The accumulation of ROS is an important cause of PD. Therefore, we propose that EAATs and VGLUTs should be important components in the study of PD. We should therefore study these transporters as they relate to PD and by doing so improve the lives of patients with PD.

## Author Contributions

XL and FZ were responsible for the study concept and design and provided a critical revision of the manuscript for important intellectual content. WW and JY drafted the manuscript. All the authors read and approved the final version.

## Conflict of Interest

The authors declare that the research was conducted in the absence of any commercial or financial relationships that could be construed as a potential conflict of interest.
